# Low PCA3 expression is a marker of poor differentiation in localized prostate tumors: exploratory analysis from 12,076 patients

**DOI:** 10.18632/oncotarget.15133

**Published:** 2017-02-07

**Authors:** Mohammed Alshalalfa, Gerald W. Verhaegh, Ewan A. Gibb, Maria Santiago-Jiménez, Nicholas Erho, Jennifer Jordan, Kasra Yousefi, Lucia L.C. Lam, Tyler Kolisnik, Jijumon Chelissery, Roland Seiler, Ashley E. Ross, R. Jeffrey Karnes, Edward M. Schaeffer, Tamara T. Lotan, Robert B. Den, Stephen J. Freedland, Elai Davicioni, Eric A. Klein, Jack A. Schalken

**Affiliations:** ^1^ GenomeDx Biosciences Inc., Vancouver, BC, Canada; ^2^ Department of Urology, Radboud University Medical Center, Nijmegen, The Netherlands; ^3^ Radboud Institute for Molecular Life Sciences, Nijmegen, The Netherlands; ^4^ Department of Urologic Sciences, University of British Columbia, Vancouver, BC, Canada; ^5^ James Buchanan Brady Urological Institute, Johns Hopkins Hospital, Baltimore, MD, USA; ^6^ Department of Urology, Mayo Clinic, Rochester, MN, USA; ^7^ Department of Urology, Feinberg School of Medicine, Northwestern University, Chicago, IL, USA; ^8^ Department of Pathology and Oncology, Johns Hopkins School of Medicine, Baltimore, MD, USA; ^9^ Sidney Kimmel Cancer Centre, Thomas Jefferson University, Philadelphia, PA, USA; ^10^ Department of Surgery, Division of Urology, Center of Integrated Research on Cancer and Lifestyle, Samuel Oschin Comprehensive Cancer Center, Cedars Sinai Medical Center, Los Angeles, CA, USA; ^11^ Glickman Urological & Kidney Institute, Cleveland Clinic, Cleveland, OH, USA

**Keywords:** prostate cancer, PCA3, initial biopsy, prognosis, under-diagnosis

## Abstract

**Background:**

Prostate cancer antigen 3 (*PCA3*) is a prostate cancer diagnostic biomarker that has been clinically validated. The limitations of the diagnostic role of *PCA3* in initial biopsy and the prognostic role are not well established. Here, we elucidate the limitations of tissue *PCA3* to predict high grade tumors in initial biopsy.

**Results:**

*PCA3* has a bimodal distribution in both biopsy and radical prostatectomy (RP) tissues, where low *PCA3* expression was significantly associated with high grade disease (p<0.001). *PCA3* had a poor performance of predicting high grade disease in initial biopsy (GS≥8) with 55% sensitivity and high false negative rates; 42% of high Gleason (≥8) samples had low *PCA3*. In RP, low *PCA3* is associated with adverse pathological features, clinical recurrence outcome and greater probability of metastatic progression (p<0.001).

**Materials and Methods:**

A total of 1,694 expression profiles from biopsy and 10,382 from RP patients with high risk tumors were obtained from the Decipher Genomic Resource Information Database (GRIDTM)prostate cancer database. The primary clinical endpoint was distant metastasis-free survival for RP and high Gleason grade for biopsy. Logistic regression analyses and Cox proportional hazards models were used to evaluate the association of *PCA3* with clinical variables and risk of metastasis.

**Conclusions:**

There is high prevalence of high grade tumors with low *PCA3* expression in the biopsy setting. Therefore, urologists should be warned that using *PCA3* as stand-alone test may lead to high rate of under-diagnosis of high grade disease in initial biopsy setting.

## INTRODUCTION

Prostate cancer (PCa) accounts for approximately 23% of all male cancers, and 9% of all cancer deaths among men in Europe and in Northern America [1]. Only a subset of these patients may harbor aggressive disease with potential recurrence after first-line treatment. To improve patient management, it is essential to use accurate, quantitative tools (i.e genomic biomarkers) to identify patients with aggressive disease. Several recent studies have demonstrated the utility of protein coding and non-coding genes as prognostic and diagnostic biomarkers [2–4]. In particular, a number of long non-coding RNAs (lncRNAs) including Prostate cancer antigen 3 (*PCA3*), *PCATs* and *SCHLAP1* have emerged as attractive diagnostic or prognostic biomarkers [3, 5, 6].

*PCA3* is over-expressed in 95% of prostate cancers, with up to 100-fold up-regulation compared to adjacent non-neoplastic cells [7]. *PCA3* is also over-expressed in high-grade prostatic intraepithelial neoplasia [8–10]. In clinical practice, urine-based *PCA3* testing has been shown to outperform the diagnostic ability of Prostate Specific Antigen (PSA) in men with a prior negative biopsy [11, 12] resulting in FDA approval for clinical use for this indication [13]. Urinary *PCA3* showed similar performance in initial biopsy setting [14–16]. However, the diagnostic performance of *PCA3* in initial biopsy setting has led to high rate of under-diagnosis of high grade disease where *PCA3* had a sensitivity of 42% with a high false negative rate using a cut-off of 60 [16]. In 463 european men scheduled for repeat biopsy, 27 (21%) cancers with a Gleason score of 7–9 would have been missed using a cut-off of 35 [11]. Similarly, *PCA3* failed to predict high grade cancers with magnetic resonance imaging (MRI) suspicion score (mSS4-5) in the initial biopsy [17]. These studies illuminate the potential limitations of using urinary *PCA3* as a diagnostic biomarker and suggest that some patients with low *PCA3* may harbor aggressive disease at initial biopsy.

In the background of the established clinical role of *PCA3*, the limitations of *PCA3* in the initial biopsy setting and the clinical and prognostic utility of tissue *PCA3* in radical prostatectomy (RP) setting has not been reported in large cohorts. In this study, we quantify the expression of *PCA3* in a large (n=12,076) localized prostate cancer cohort and relate these data to clinicopathological parameters, generating, to our knowledge, the largest study that correlates *PCA3* expression to prostate cancer clinical outcome.

## RESULTS

### Expression of *PCA3* in primary prostate cancer tissue

**Figure 1:**
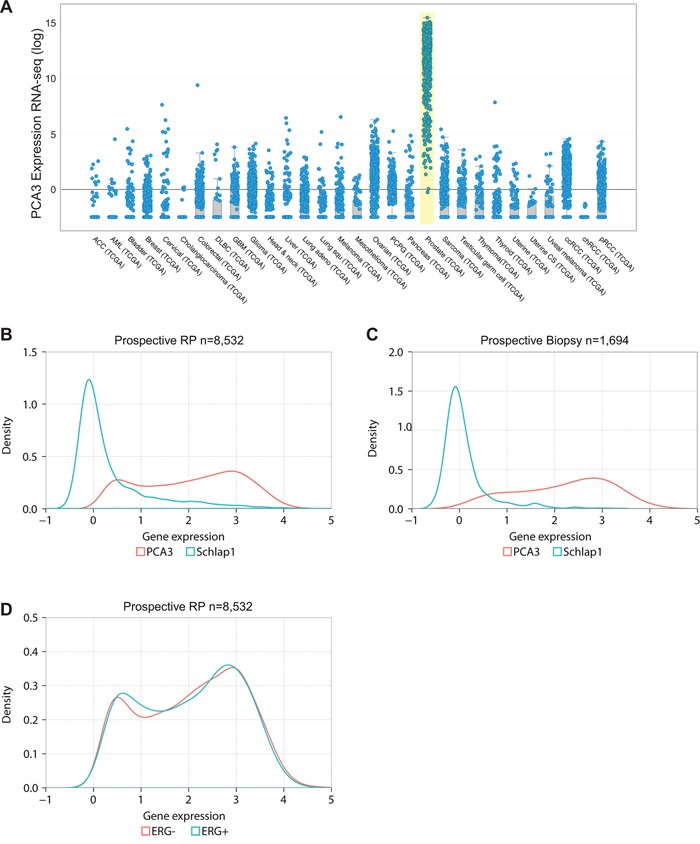
Pan-cancer and prostate-specific expression of *PCA3* **A.** TCGA Pan cancer analysis showing *PCA3* to be PCa specific. **B.** Distribution of *PCA3* (red) and SChLAP1 (blue) expression in prospective RP (B) and in biopsy **C.** showing that *PCA3* has a bimodal distribution unlike SChLAP1 that is right skewed. **D.** Distribution of *PCA3* in ERG+ and ERG- tumors as determined by our group previously [19] in prospective RP cohort.

Previous studies have evaluated *PCA3* in urine [11, 12], yet the characteristics of *PCA3* expression in prostate tissue remain under studied [18]. To begin to explore the prostate-specific expression of *PCA3*, we evaluated publically available The Cancer Genome Atlas (TCGA) data finding that while *PCA3* was expressed in very few samples from other cancers, it was most abundant in prostate cancer (Figure 1A).

To expand our initial observations regarding the expression of *PCA3* in prostate cancer, we used an array-based approach to profile a large cohort of 8,532 and 1,694 prostate tumors from prospective RP and biopsy tissues, respectively. For this analysis, we included the well-characterized lncRNA *SCHLAP1* as control for high grade prostate cancer [3]. As expected, we observed a strong right skewed distribution for *SCHLAP1*, confirming the reported expression pattern of this gene in prostate cancer (Figure 1B) [3]. In stark contrast, *PCA3* demonstrated a bimodal distribution where 23% and 29% of patients were categorized as having low *PCA3* (≤ 1.3) in biopsy and RP samples respectively (Figure 1B-1C). Gene with bimodal distribution exhibit mainly either high or low expression with small proportion with intermediate expression, unlike normal distribution in which most patients express intermediate expression. This observation was validated using a retrospective cohort of 1,850 patient expression profiles from the Decipher GRID database (Supplementary Figure 2). This bimodality could be a result of tumor cells going through a differentiation process and change of cellular state.

To interpret this observation in the context of prostate cancer genomics, we hypothesized that ERG fusion may be driving the expression of *PCA3*. However, *PCA3* displayed a bimodal distribution in both ERG+ and ERG- tumors, as determined previously by our group [19], suggesting that the observed bimodal distribution is independent of ERG expression (Figure 1D).

### High grade tumors in biopsy tissues have low *PCA3* expression

**Figure 2:**
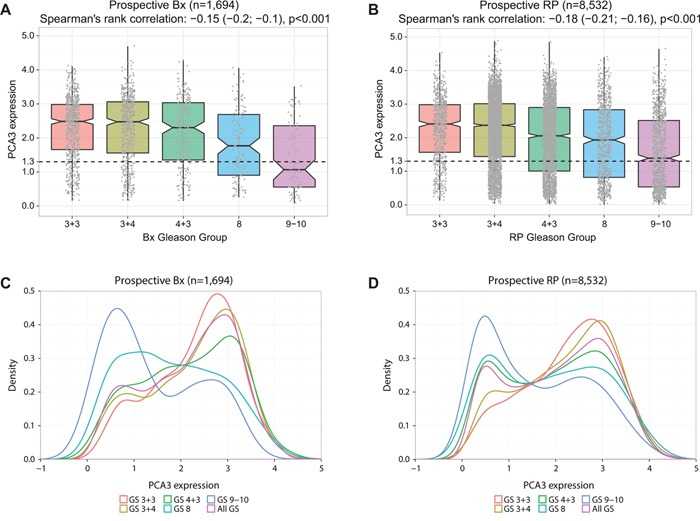
Associations of *PCA3* expression and pathological characteristics at RP **A.** *PCA3* expression across Gleason groups (1-5) in biopsy samples (n=1,694) and **B.** RP samples (n=8,532) showing that around 50% of Gleason 5 group have low *PCA3*. **C-D.** Distribution of *PCA3* across Gleason groups in biopsy (C) and RP samples (D) showing GS5 to be more enriched with low *PCA3* while GS1 enriched more with high *PCA3*.

To interpret the bimodal distribution clinically, we first investigated if *PCA3* bimodality related to Gleason score in 1,694 initial biopsy samples from the Decipher GRID database. We found *PCA3* was significantly associated with Gleason group (Spearman's correlation: -0.15, p<0.001) where high Gleason group (Group 4&5) tumors showing lower expression of *PCA3* (Figure 2A). Out of 234 samples with Gleason group 4&5, 105 (44.8%) had low *PCA3* levels which was significantly higher than 18% (out of 1228) Gleason groups (1&2) (Fisher's test p <0.001). Out of 93 samples with Gleason group 5, the percentage of low *PCA3* reached 58%. These results indicate that *PCA3* is a poor predictor of high grade disease in biopsy with sensitivity of 55% and false negative rate of 44.8% in high grade tumors.

### Low *PCA3* in RP tissues is associated with adverse pathological variables

Next, we investigated if low *PCA3* relates to clinicopathological variables at RP. In the prospective cohort (n=8,532) and pooled retrospective cohort (n=1,850), we found low *PCA3* to be associated with high Gleason grades (groups 4&5) (Spearman's correlation: -0.18, p<0.001) (Figure 2B). In the prospective cohort, out of 1,790 patients in the Gleason groups 4&5, 763 (42%) have low *PCA3*. These results confirmed high levels of *PCA3* was a poor predictor of high grade disease and low *PCA3* levels was not an indication of the absence of cancer.

Distribution plots of *PCA3* across Gleason groups showed Gleason groups 1 and 2 had a right modal distribution for *PCA3*, while group 5 (GS 9-10) had left modal distribution, in both the prospective biopsy (Figure 2C), prospective RP (Figure 2D) and retrospective RP cohorts (Supplementary Figure 3). The distributions for GS 4+3 and GS 8 were bimodal, however, the bimodal distribution for GS 3+4 was skewed to the right (Figure 2C). These data suggest the expression of *PCA3* expression in prostate tissue may reflect the differentiation status of tumors, correlated with the amount of pattern 4 tumor present in tumors with mixed Gleason grade.

Next, we explored the association of *PCA3* expression (low vs. high) with pathological tumor characteristics, including pathological stage and Gleason group (Table 1). In the prospective, Gleason group 5 (Odd Ratio (OR): 3.88, 95% Confidence Interval (CI): 3.09-4.86, p<0.001) when compared to group 1, Seminal Vesicle Invasion (SVI) (OR: 2.12 [1.89-2.39], p<0.001), Extra Prostatic Extension (EPE) (OR: 1.59 [1.44-1.75], p<0.001) and Lymph Node Invasion (LNI) (OR: 2.14 [1.72-2.66], p<0.001) were significantly associated with low *PCA3* on Univariable Analysis (UVA). Similar results were obtained using the retrospective cohort (Table 1). Using Multivariable Analysis (MVA) logistic regression, EPE, SVI and Gleason group 5 remained significantly associated with *PCA3* expression in the prospective and retrospective cohorts (Table 1). The negative correlation between *PCA3* expression and Gleason scores was also observed in urinary samples (n=50) (Supplementary Figure 4) where *PCA3* expression was measured using the same Human Exon arrays used for *PCA3* expression measurement in RP and biopsy tissues.

**Table 1:** Univariable and multivariable analysis associating clinicopathologic risk factors with PCA3 in prospective (n=8,532) and retrospective cohorts (n=1,850)

	Prospective data (n=8,532)			
	UVA		MVA	
	Estimate(95% CI)	p value	Estimate (95% CI)	p value
pre-PSA (ref: <10 ng/ml)	1.21 (1.05-1.4)	0.008	0.91 (0.78-1.07)	0.26
EPE	1.59 (1.44-1.75)	<0.001	1.24 (1.07-1.43)	0.005
SVI	2.12 (1.89-2.39)	<0.001	1.61 (1.35-1.92)	<0.001
SM	0.98 (0.89-1.08)	0.73	0.97 (0.85-1.12)	0.69
LNI	2.14 (1.72-2.66)	<0.001	1.42 (1.05-1.91)	0.02
Gleason group 2 (ref: group 1)	1.21 (0.99-1.49)	0.07	1.1 (0.77-1.57)	0.6
Gleason group 3 (ref: group 1)	1.96 (1.59-2.41)	<0.001	1.58 (1.1-2.27)	0.01
Gleason group 4 (ref: group 1)	2.33 (1.83-2.96)	<0.001	1.83 (1.23-2.72)	0.003
Gleason group 5 (ref: group 1)	3.88 (3.09-4.86)	<0.001	2.83 (1.93-4.15)	<0.001
	**Pooled retrospective data (n=1,850)**			
	**UVA**		**MVA**	
	**Estimate (95% CI)**	**p value**	**Estimate (95% CI)**	**p value**
pre-PSA (ref: <10 ng/ml)	0.9359(0.756-1.158)	0.542	0.8036(0.638-1.012)	0.064
EPE	1.3662(1.112-1.679)	0.003	1.1358(0.901-1.433)	0.282
SVI	1.6435(1.312-2.059)	<0.001	1.3382(1.031-1.737)	0.028
SM	0.8033(0.659-0.979)	0.030	0.7931(0.640-0.983)	0.035
LNI	1.8508(1.353-2.532)	<0.001	1.2657(0.887-1.807)	0.194
Gleason group 2&3 (ref: group 1)	0.9361(0.699-1.253)	0.657	0.9123(0.651-1.278)	0.593
Gleason group 4 (ref: group 1)	1.8648(1.272-2.734)	0.001	1.7886(1.177-2.718)	0.006
Gleason group 5 (ref: group 1)	2.1513(1.542-3.001)	<0.001	1.951(1.320-2.883)	<0.001

pre-PSA: preoperative prostate specific antigen; EPE: extra prostatic extension; SVI: seminal vesicle invasion; SM: surgical margins; LNI: lymph node invasion.

### Low *PCA3* expression tracks with poor prognosis and metastasis after RP

**Figure 3:**
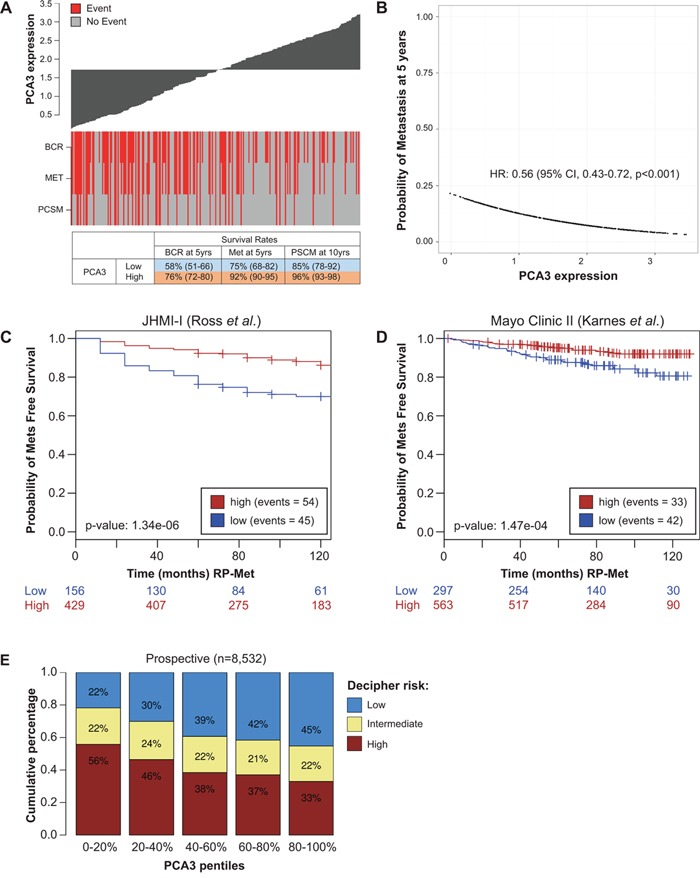
Prognostic impact of *PCA3* in localized prostate tumors **A.** Waterfall plot of *PCA3* with clinical endpoint annotations. BCR, biochemical recurrence; MET, metastasis, PCSM, PCa-specific mortality. **B.** Association between probability of 5-year metastasis and *PCA3* expression in 665 samples pooled from two cohorts [21, 22] and the subcohorts from two case cohorts (JHMI-I, Mayo-Clinic II) [20, 23]. **C-D.** Kaplan Meier analysis in two case-cohorts from JHMI-I and Mayo-Clinic II. *PCA3* expression was categorized into low vs high. **E.** Association between Decipher risk categories and *PCA3* levels showing low *PCA3* patients are more enriched with high risk Decipher.

To assess the association of *PCA3* expression with clinical endpoints we plotted *PCA3* expression in a John Hopkins Medical Institute (JHMI) cohort previously used to validate the Decipher test (JHMI-I) [20] and tagged events for biochemical recurrence (BCR), metastasis (MET) and Prostate Cancer Specific Mortality (PCSM) free survival (Figure 3A). Here, we observed patients with low *PCA3* expression had lower 5-year BCR free survival rates (58%), 5-year MET free survival rates (75%) and 10-year PCSM free survival (85%) compared to high *PCA3* patients in JHMI-I cohort (76, 92, 96% for BCR, MET and PCSM respectively) (Figure 3A, Supplementary Table 3). A similar trend was observed in the Mayo-Clinic II cohort that was used to validate Decipher test (Supplementary Table 3).

In a set of 665 patients pooled from two cohorts [21, 22] and the subcohorts of two previously published case cohorts [20, 23], we found patients with high *PCA3* had nearly 50% lower risk of developing metastasis at 5-years, compared to those with low *PCA3* (Hazard Ratio (HR): 0.56 (95% CI: 0.43-0.72, p<0.001) (Figure 3B). Kaplan-Meier analysis showed that low *PCA3* is significantly associated with early metastasis after radical prostatectomy in both the JHMI-I (HR: 0.35, 95% CI: 0.22-0.57, p<0.001) and Mayo-Clinic II (HR: 0.45, 95% CI: 0.26-0.79, p=0.005) datasets (Figure 3C, 3D). In the prospective RP cohort outcome data are currently not available, making it difficult to assess the prognostic impact of *PCA3*. However, in previous work we have demonstrated a robust correlation between Decipher risk categories and metastasis [20]. As such, we used the frequency of Decipher risk categories as a surrogate for metastasis (low, intermediate vs high risk) across *PCA3* expression pentiles (5 categories of *PCA3* expression each with 20% of samples). Using these metrics, we observed 56% and 33% of patients with high Decipher showed low and high *PCA3* expression, respectively (Fisher's test, p<0.001) (Figure 3E). Interestingly, one of the top positively correlated genes across 8,532 samples is PCAT-14 (R=0.32, p<0.0001), which has also a bimodal distribution (Supplementary Figure 5) and has shown to increase migration [5]. Additional analysis found genes negatively correlated to *PCA3* expression were enriched in gene sets related to epithelial-to-mesenchymal transition (EMT) based on Gene Set Enrichment Analysis (Supplementary Figure 6). Collectively, these data provide supporting evidence indicating lower *PCA3* levels correlate with a higher risk of metastasis and more aggressive prostate cancer at RP and biopsy setting.

## DISCUSSION

The clinical utility of the *PCA3* has been established as an independent diagnostic biomarker to predict prostate cancer in initial and repeat biopsy setting, primarily through urine-based analyses [6, 14]. However, the performance of *PCA3* in initial biopsy setting has been shown in several studies to be poor with high false negative rate and low sensitivity rate leading to under-diagnosis of high grade disease [11, 16, 24]. Additionally, establishing the prognostic significance of the same assay has been challenging, in part due to small cohort sizes.

In this study, we utilize the large collection of biopsy and RP expression profiles from the Decipher GRID database (GenomeDx Biosciences Inc.) to study *PCA3* expression and link it to clinical data and differentiation levels of tumor. We found that *PCA3* demonstrated a striking bimodal distribution in prostate tissues representing distinct differentiation levels, which was not associated with ERG status and is distinct from other prostate specific prognostic lncRNA (i.e SChLAP1) that is right skewed. These data suggest *PCA3* expression may be indicating two distinct differentiation cellular or tissue states within the prostate tumor; an early state where high levels of *PCA3* are detectable and a second, late stage state where *PCA3* levels decline. The bimodal expression pattern suggests unique biology for *PCA3* which is not consistent with a ramping up of expression, as was observed for *SCHLAP1*. This observation motivated us to further understand the biology and clinical implication of *PCA3* cycle.

We have associated *PCA3* with Gleason scores in large biopsy and RP cohorts from the Decipher GRID and found patients with high Gleason have lower *PCA3* expression (42-45%). These results indicate that most patients with high Gleason would have been reported negative on diagnostic biopsy if *PCA3* biomarkers was used for initial diagnosis. Moreover, in the prospective cohort from Decipher GRID (RP, biopsy) where metastatic outcome was not available, we found that low *PCA3* was strongly associated with high risk Decipher scores and was a surrogate for metastasis. Additional analyses showed that genes negatively correlated with *PCA3* are associated with migration pathways such as EMT. Based on expression correlation, *PCAT-14* was the top correlated genes. In a recent report [5], *In-vitro* analysis showed that low *PCAT-14* expression increased migration while overexpressing *PCAT-14* reduced cellular growth, migration, and invasion. These results suggest that patients with low *PCA3* are at a significantly higher risk of developing metastasis after RP.

Recent work [25, 26] proposed that targeting *PCA3* may be a putative therapeutic option to inhibit PCa growth based on interfering siRNA in LNCaP and PC3 cells. These results are contradicting our observation possibly due the different nature of genomics of LNCaP cells and RP/biopsy tissues (used in this cohort) and different AR-activity in these specimens. We observed strong positive correlation between *PCA3* and *ABCC4*, *KLK2* and *KLK3* that are AR target genes supporting the modulatory effect between *AR* and *PCA3*. Activated androgen receptor (AR) also strongly induces *PCA3* transcription [26, 27]. We believe that the interaction between *AR*-axis induced by testosterone and *PCA3* may significantly elucidate the functional impact of *PCA3*. Low pre-treatment free testosterone levels, are significantly associated with tumor grade and stage, with lower testosterone levels in patients with high grade PCa [27]. Prostate cancer patients with lower circulating free testosterone levels also have poor prognosis factors and higher tumor burden prior to treatment [28]. These findings reinforce the idea that low *PCA3* levels in high grade PCa may reflect lower circulating androgen levels, leading to weaker *AR* activation and transcriptional activity. This suggests that *PCA3* may act as a surrogate marker for serum testosterone.

Based on results presented in our study, *PCA3* assays as currently formatted have limited utility in detecting men with higher grade disease. Even though such tools have shown to reduce number of biopsies, they have high rate of missing high grade disease due to low *PCA3* levels. We have observed such a case recently, where a patient was negative for prostate cancer as assessed by urinary *PCA3*, but was later diagnosed to have very high grade disease (GS 9) and high Decipher metastasis risk. To overcome this limitation, recently developed commercial diagnostic tools have incorporated additional biomarkers like ERG [7], HOXC6, DLX1 [28]. Additional markers designed to detect higher grade disease and non-ERG tumors would likely improve the utility of the assay. This study suggests that next-generation diagnostic tools using *PCA3* and ERG should incorporate additional biomarkers to ensure high grade disease is detected in low *PCA3* and ERG-fusion negative samples.

The current study has some limitations. We acknowledge the majority of our analyses are based on tissue *PCA3* whilst current *PCA3* assays are urine based. We have generated preliminary data profiling gene expression from a small number of urine samples (n=50) using the same Human Exon arrays (Supplementary Figure 4). Even though the range of *PCA3* expression is different in tumor tissue and urine samples, the *PCA3* trend in urine sample is very similar to tissue *PCA3* suggesting that results from this study may hold true when conducted in large number of urine sample. Additional studies are warranted to firmly establish the relationship between high grade prostate cancer and the utility of *PCA3* score in urine-based diagnostic tests. Additional urine-based diagnostic tools that incorporates additional biomarkers are needed to overcome limitations of *PCA3* assays.

## MATERIALS AND METHODS

### Study population

A total of 10,382 expression profiles from RP tissues, primarily with adverse pathology and other high risk features, and 1,694 profiles from initial biopsy tissues were obtained from the Decipher GRID™ prostate cancer database (NCT02609269). This cohort represents cases from multi-institutional retrospective cohorts (Supplementary Table 1) [2, 20, 21, 23, 29] with detailed clinical, pathological, treatment and outcomes data and de-identified, anonymized prospective cases with basic demographic and pathological data from clinical use of the Decipher test from RP and biopsy settings (Supplementary Table 2).

### Tissue processing, microarray data preprocessing and PCA3 expression measurement

Specimen selection, RNA extraction, and Human Exon 1.0 ST Array hybridization was done in a Clinical Laboratory Improvement Amendments (CLIA/CAP)-certified laboratory facility (GenomeDx Biosciences, San Diego, CA, USA) as previously described [30]. The Supplementary Material provides additional details. *PCA3* gene expression was summarized using the mean of the 5 probe sets in the Human Exon 1.0 ST array that are falling in the *PCA3* locus (Supplementary Figure 1) and corrected for batch effects using an empirical Bayes framework (ComBat in “sva” R package).

### Statistical analysis

Statistical analyses were performed in R v3.2.2, and all tests were two-sided using a 5% significance level. Clinical variables were categorized as follows: Gleason Score (GS) was grouped into ISUP groups: Group 1(GS 6), Group 2(3+4), Group 3(4+3), Group 4(8), Group 5 (9-10). Pre-operative PSA was categorized into low (≤10 ng/ml) and high (>10 ng/ml) groups. The following variables were binary (present vs. absent): Extra-prostatic extension (EPE), seminal vesicle invasion (SVI), surgical margins (SM) and lymph node invasion (LNI). Fisher's exact test was used to study the association between categorical variables. *PCA3* expression was dichotomized using the optimal cut-off value splitting *PCA3* into low (≤1.3) vs high (>1.3) using the “optimize R” package that is based on combination of golden section search and successive parabolic interpolation. Correlations of *PCA3* expression with pathologic stage and pathologic Gleason score were computed using Spearman's rank correlation. Univariable (UVA) and multivariable (MVA) logistic regression analyses were used to evaluate the association of *PCA3* and clinical variables. Kaplan Meier curves stratified by *PCA3* expression levels (low vs. high) were constructed to obtain metastasis-free survival rates after RP. Cox proportional hazards analysis were used to determine the performance of *PCA3* in predicting risk of metastasis. In time to event analyses, event times were defined as the time from RP to metastasis. Decipher risk categories, an extensively validated measure of prostate cancer metastasis risk [20, 22, 23, 29–32], were used as a surrogate for metastasis (low, intermediate vs high risk) in the prospective cohort where no metastasis outcomes were available.

## CONCLUSION

In conclusion, we report that high levels of *PCA3* may be a poor predictor of high grade disease in the initial biopsy setting and low *PCA3* expression in primary tumors may portend a poor prognosis. We observed a unique bimodal distribution for *PCA3*, where low levels of *PCA3* were associated with high grade disease in urine, tissue biopsy, and tissue RP, and poor survival and increased rates of metastasis after RP. To our knowledge, this is the largest study characterizing the associations between *PCA3* RNA from RP tissues with pathological variables at RP and clinical outcome. Results in this study suggest that sole use of *PCA3* as a stand-alone marker for prostate cancer may provide false negatives for patients with higher grade disease and urologists should be warned of this limitation.

## SUPPLEMENTARY MATERIALS METHODS FIGURES AND TABLES


