# Supplementary material for: Low PCA3 expression is a marker of poor differentiation in localized prostate tumors: exploratory analysis from 12,076 patients
Source: Oncotarget. 2017 Feb 7;8(31):50804–13. doi: 10.18632/oncotarget.15133 (PMC5584206; doi:10.18632/oncotarget.15133)
Supplement: Supplementary file 1 [file oncotarget-08-50804-s001.pdf]

Low *PCA3* expression is a marker of poor differentiation in localized prostate tumors: exploratory analysis from 12,076 patients

SUPPLEMENTARY MATERIALS AND METHODS

Study cohort

The retrospective set of 1,850 tumor tissues from radical prostatectomy (RP) expression profiles with clinical follow up and outcomes data were pooled from five institutions (Mayo Clinic I & 2: n=777, Thomas Jefferson: n=133, Cleveland Clinic: n=182, John Hopkins: n=638, Durham VA: n=120) that were from previously reported studies [Supplementary Table 1]. Most of these patients have clinical variables and metastatic outcome follow-up up to 13 years. The prospective set of 8,532 tumor tissues from RP and 1,694 from biopsy expression profiles from de-identified and anonymized Decipher Prostate Cancer Classifier were retrieved from the GRID. These profiles had associated pathological variables, but no outcomes data.

Total RNA was extracted and purified using the RNeasy FFPE kit (Qiagen, Valencia, CA). RNA was amplified and labeled using the Ovation FFPE WTA system (NuGen, San Carlos, CA) and hybridized to Human Exon 1.0 ST GeneChips (Affymetrix, Santa Clara, CA). Quality control was performed using Affymetrix Power Tools [15], and normalization was performed using the Single Channel Array Normalization (SCAN) algorithm [16].

Enrichment analysis

Genes were ranked based on their Pearson's correlation to *PCA3* in the prospective cohort and gene set enrichment analysis (GSEA) was carried out to identify gene sets associated with *PCA3* by using the GSEA analysis tool (version 2.2.0) downloaded from the Broad Institute website (http://www.broadinstitute.org/gsea). The curated gene sets of the Molecular Signature Database (MSigDB) version 4.0 was used for enrichment. The false discovery rate (FDR) for GSEA is the estimated probability that a gene set with a given normalized enrichment score (NES) represents a false-positive finding, and an FDR <0.25 is considered to be statistically significant for GSEA.

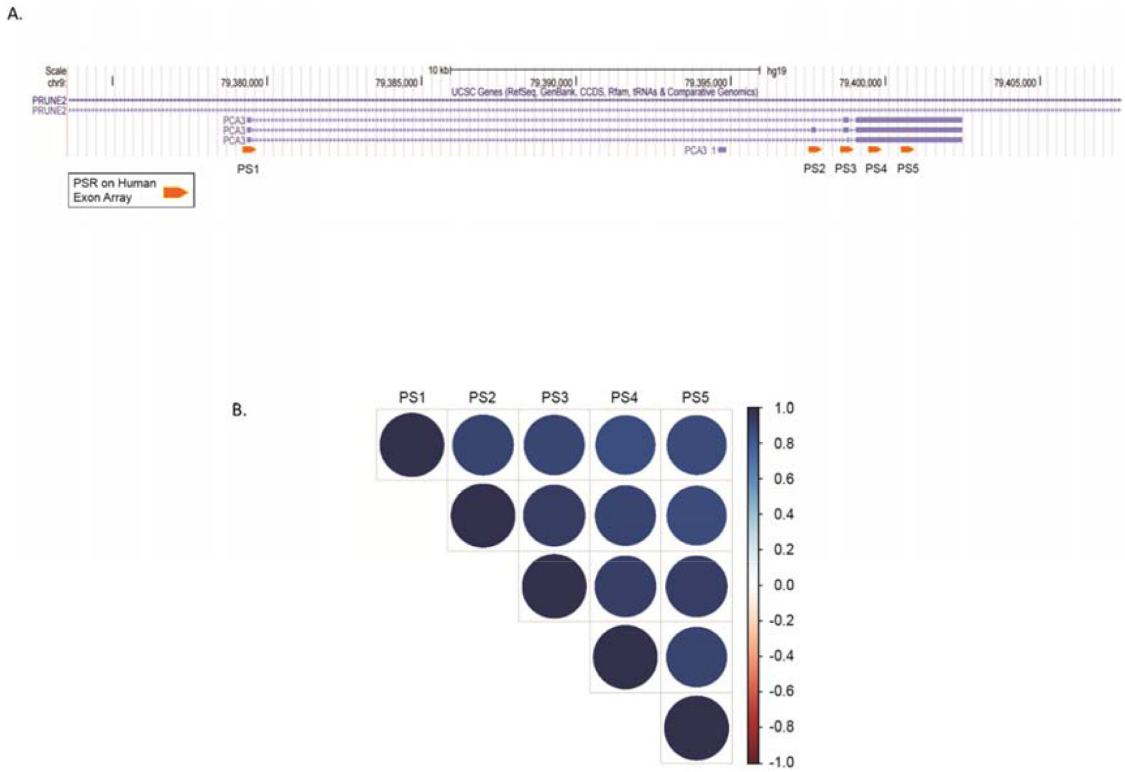

A. Localization of the 5 PCA3 probe selection regions (PSRs) covering most of the exonic regions based on the Human Exon 1.0ST assay. B. Pair wise Pearson correlation analysis of the 5 PCA3 PSRs.

Supplementary Figure 1: Localization of the 5 PCA3 probe sets and their pairwise correlation. A. localization of the 5 PCA3 Probe set regions (PS1-5) covering most of the exonic regions based on the Human Exon 1.0ST Array. **B.** Pair-wise Pearson's correlation among the 5 PS showing high correlation among them.

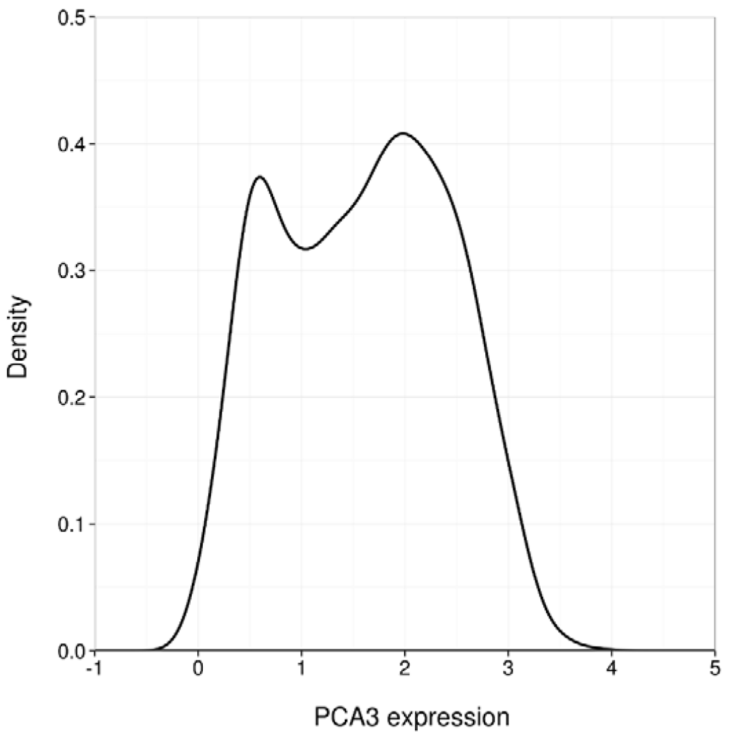

Supplementary Figure 2: Bimodal distribution of PCA3 in pooled retrospective cohorts (n=1,850).

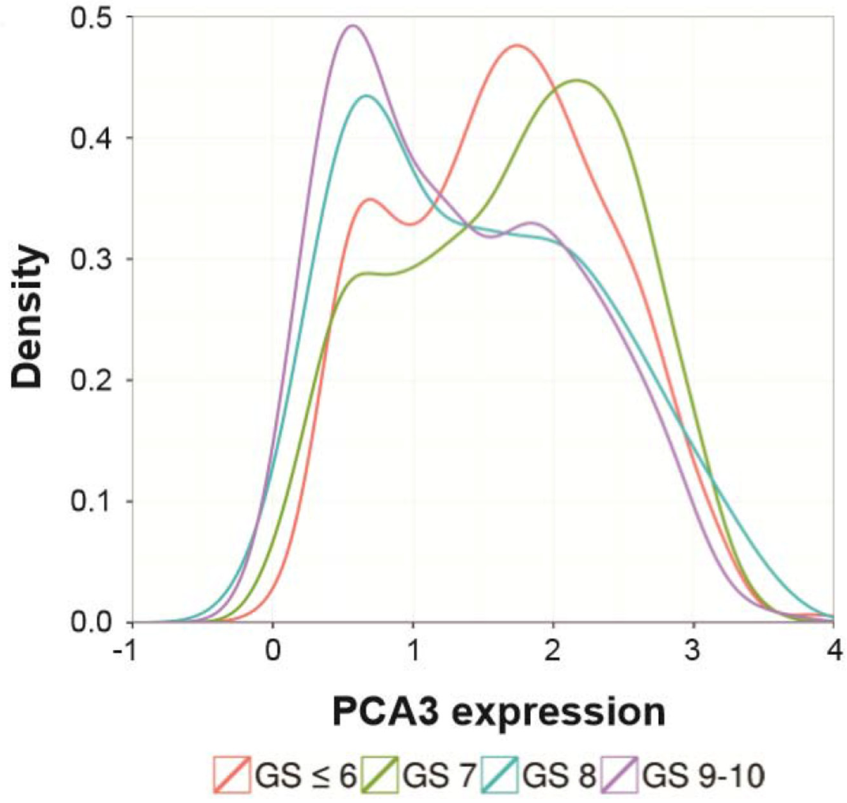

Supplementary Figure 3: PCA3 distribution in retrospective cohorts (m=1,850).

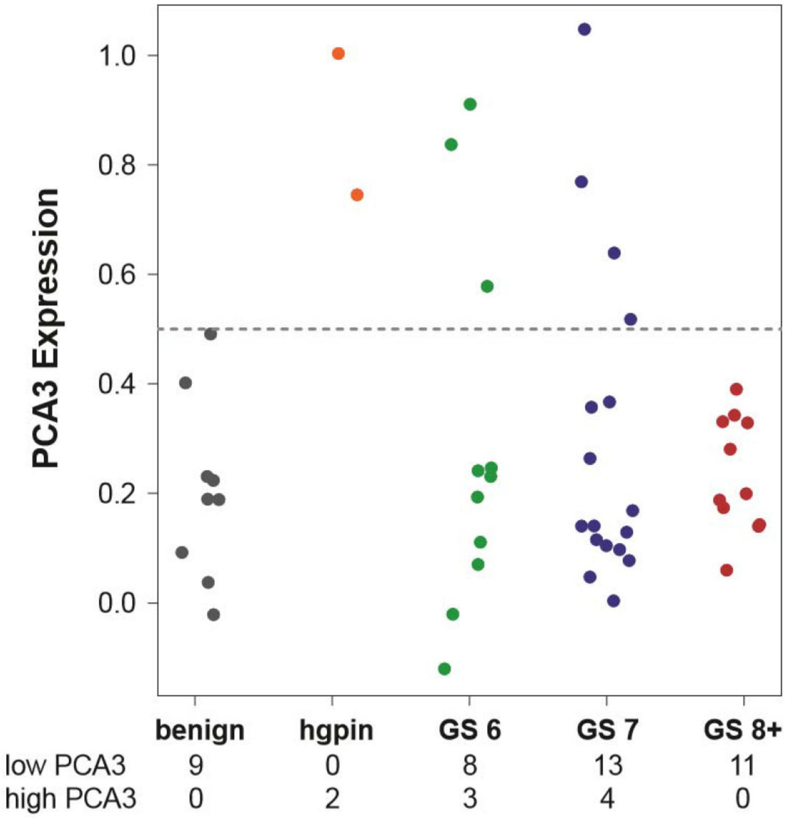

Supplementary Figure 4: PCA3 expression from urine samples profiles using the same Human Exon arrays.

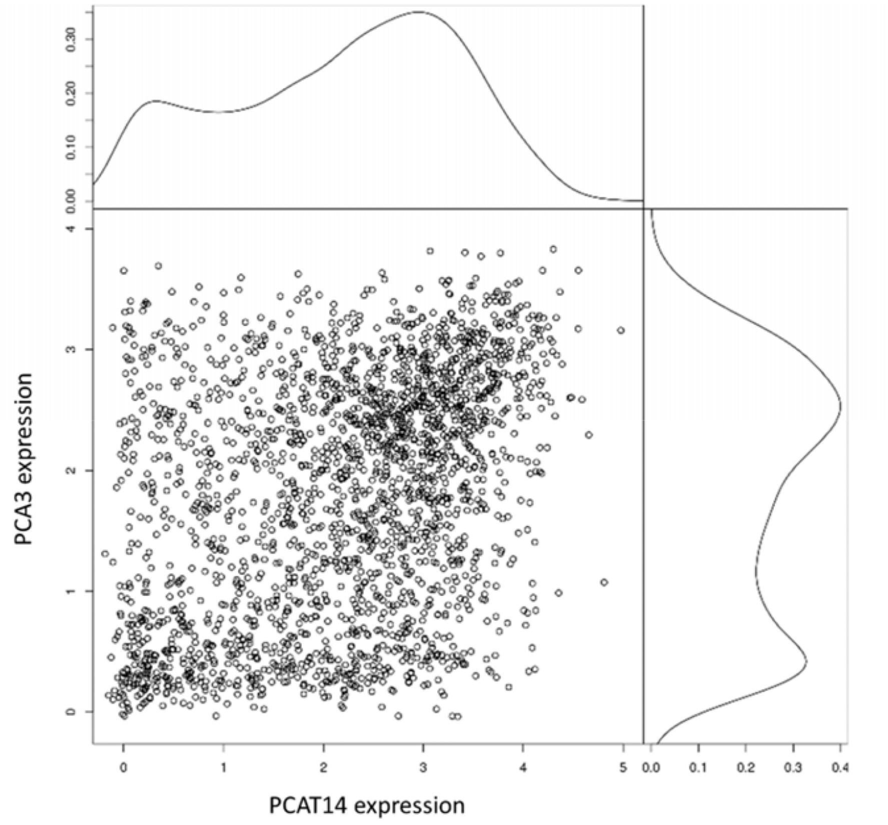

Supplementary Figure 5: Correlation between PCA3 and top positively correlated gene (PCAT14).

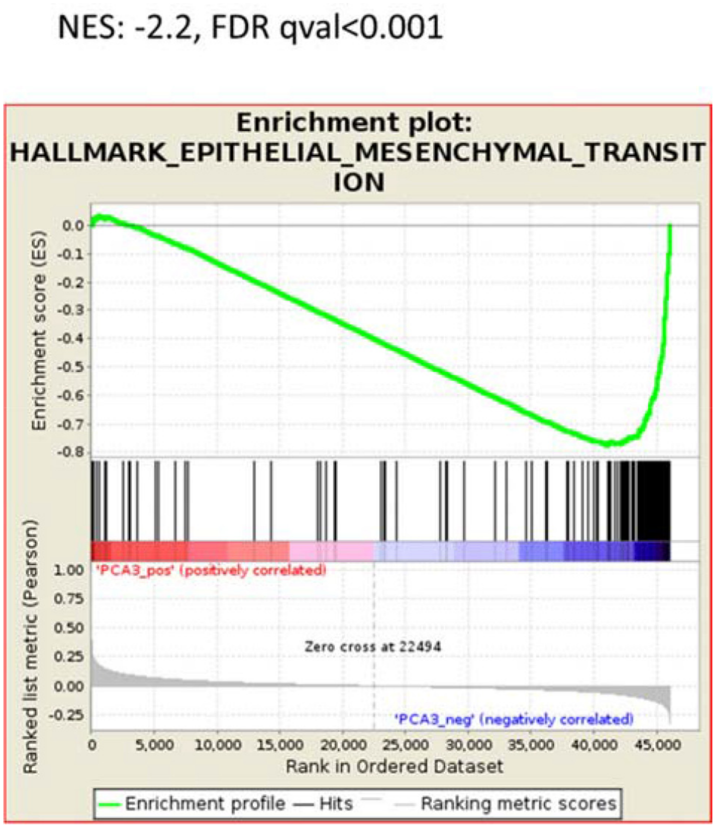

Supplementary Figure 6: Gene Set Enrichment Analysis showing genes negatively associated with PCA3 are associated with EMT.

Supplementary Table 1: Prostate cancer cohorts used for genome wide expression profiling

Data set	Tissue	Study years	Patients (n)	Reference [PMID]	GEO ID	Profiling assay
Thomas Jefferson University	FFPE	1997-2009	133	Den *et al* [25035207]	GSE72291	Decipher assay
Cleveland Clinic	FFPE	1990-2009	182	Klein *et al* [25466945]	GSE62667	Decipher assay
John Hopkins RP	FFPE	1992-2010	358	Ross *et al* [26058959]	NA	Decipher assay
Mayo Clinic II	FFPE	2000-2006	232	Karnes *et al* [23770138]	GSE62116	Decipher assay
Mayo Clinic I	FFPE	1987-2001	545	Erho *et al* [23826159]	GSE46691	Decipher assay
Durham	FFPE	1990-2010	120	Freedland *et al* [26806658]	NA	Decipher assay
John Hopkins AAM	FFPE	1992-2010	136	Faisal *et al* [26443432]	NA	Decipher assay
Johns Hopkins	FFPE	2001-2009	144	Trock *et al* (abstract)	NA	Decipher assay
Prospective biopsy	FFPE	2016	1,694	NA	NA	Decipher assay
Prospective RP	FFPE	2013- 2016	8,532	NA	NA	Decipher assay

Proportion of cohort with any of the following: prostatectomy Gleason score 8-10, extraprostatic extension (EPE), seminal vesicle invasion (SVI) or lymph node involvement (LNI).

Supplementary Table 2: Characteristics of prospective biopsy and RP samples from Decipher GRID database

Variables	Prospective RP	Prospective Bx
	No. (%); median (IQR)	No. (%); median (IQR)
**Total**	8,532 (100%)	1694 (100%)
**Age (years) (at RP or Bx)**	65.5 (60, 69.2)	
**PSA at diagnosis (ng/mL)**	6.5 (4.8, 9.7)	6.3 (4.6, 9.2)
<10 ng/mL	3,788 (44.4%)	1108 (65.4%)
10-20 ng/mL	874 (10.2%)	225 (13.3%)
>20 ng/mL	298 (3.5%)	93 (5.5%)
Unknown	3,572 (41.9%)	268 (15.8%)
**Gleason grade group (Bx or post-RP)**		
Group 1 (GS 3+3)	688 (8.1%)	659 (38.9%)
Group 2 (GS 3+4)	3,695 (43.3%)	569 (33.6%)
Group 3 (GS 4+3)	2,354 (27.6%)	232 (13.7%)
Group 4 (GS 8)	770 (9%)	141 (8.3%)
Group 5 (GS 9-10)	1,020 (12%)	93 (5.5%)
Unknown	5 (0.1%)	0 (0.0%)
**Clinical stage**		
T1	.	898 (53%)
T2a	.	123 (7.3%)
T2b/c	.	121 (7.1%)
T3/4	.	18 (1.1%)
Unknown	.	534 (31.5%)
**Pathologic stage**		
pT2	3,606 (42.3%)	.
pT3a	2,752 (32.3%)	.
pT3b	1,388 (16.3%)	.
pT4	240 (2.8%)	.
Unknown	546 (6.4%)	.
**SM**		
Positive	4,213 (49.4%)	.
**EPE**		
Present	4,163 (48.8%)	.
**SVI**		
Present	1,453 (17%)	.
**LNI**		
Positive	346 (4.1%)	.

Supplementary Table 3: Survival rates of low and high PCA3 at 5 and 10 years in JHMI and Mayo Clinic II cohorts

Cohort	PCA3 levels	BCR		Metastasis		PCSM	
		Survival rate at 5 years	Survival rate at 10 years	Survival rate at 5 years	Survival rate at 10 years	Survival rate at 5 years	Survival rate at 10 years
JHMI	Low	58.1 (50.8-66.6)	48.1 (40.4-57.2)	74.8 (68.2-82.1)	68.2 (60.7-76.6)	93.8 (90.0-97.8)	85 (78.6-91.8)
	High	75.8 (71.8-80.0)	72.4 (68.1-77.0)	92.2 (89.6-94.8)	86.8 (83.2-90.5)	98.1 (96.8-99.4)	95.6 (93.4-97.9)
Mayo Clinic II	Low	63.5 (57.7-69.9)	52.7 (46.0-60.4)	89.1 (85.2-93.1)	81.4 (75.4-87.9)	95.2 (92.4-98.0)	89.1 (83.5-95.1)
	High	70.5 (66.8-74.3)	58.7 (54.2-63.6)	94.9 (93.1-96.8)	91.4 (88.6-94.2)	98.5 (97.5-99.5)	94.5 (91.5-97.5)
